# Enhanced Hepatocarcinogenicity Due to Agonists of Peroxisome Proliferator-Activated Receptors in Senescent Rats: Role of Peroxisome Proliferation, Cell Proliferation, and Apoptosis

**DOI:** 10.1100/tsw.2002.352

**Published:** 2002-05-30

**Authors:** Jihan Youssef, Mostafa Badr

**Affiliations:** University of Missouri-Kansas City, 2411 Holmes Street, M3-115, Kansas City, MO 64108-2792, USA

**Keywords:** aging, peroxisomes, peroxisome proliferator-activated receptors, apoptosis, cell proliferation, oxidative stress, liver cancer

## Abstract

Exposure to agonists of peroxisome proliferator-activated receptor alpha (PPAR*α*) causes liver cancer in rodents, with aged animals being more susceptible than their younger counterparts to this effect. Treatment with these chemicals produced a five- to sevenfold higher yield of grossly visible hepatic tumors in old rats compared to young animals. The enhanced susceptibility of the aged livers to the carcinogenic effect of PPAR agonists could not be explained by differences in levels of peroxisomal and/or cell proliferation between young and old animals, as neither of these responses was exaggerated with aging. Reported studies have shown that activating PPARa results in the suppression of hepatic apoptosis. This effect is expected to diminish the ability of the liver to purge itself of pre-existing neoplastic cells, allowing them to progress to tumors. New findings from our laboratories show that the aged liver is exceedingly sensitive to the antiapoptotic effect of PPAR agonists. In addition, aged livers showed remarkably higher levels of the antiapoptotic protein Bcl-2 than livers of young, adult, and middle-aged animals. Interestingly, the PPARa agonist Wy-14,643 significantly diminished elements of the proapoptotic machinery (e.g., Bax, caspases, and fas) in the aged liver, while remarkably increasing elements of this machinery in younger animals. Taken together, while activation of PPARs appears to inhibit apoptosis in livers of senescent animals, activating these receptors seems to stimulate the apoptotic machinery in young animals. This paradoxical effect may be responsible for the exaggerated sensitivity of the aged liver to the carcinogenic effect of agents that activate PPARs.

## INTRODUCTION

The incidence of various cancers increases with about the fifth power of age in both short-lived species, such as rats, and long-lived species, such as humans[1], clustering in the terminal part of the life span[2]. Oxidative damage to cellular macromolecules has been postulated to be a major cause of aging and cancer[1]. Among sources of endogenous oxidants are byproducts of mitochondrial, microsomal, and peroxisomal metabolism[1].

### Peroxisomes and Peroxisome Proliferator-Activated Receptors (PPARs)

Peroxisomes are respiratory organelles that play a significant role in cellular active oxygen metabolism[3]. Urate oxidase, acyl-CoA oxidase, amino acid oxidase, polyamine oxidase, trihydroxycholestanoyl-CoA oxidase, and pristanoyl-CoA oxidase are among the oxidases identified in peroxisomes[4].

During the last decade, a group of nuclear receptors belonging to the steroid hormone receptor superfamily has been identified as a mediator of the pleiotropic responses to a myriad of chemicals known collectively as peroxisome proliferators[5,6,7]. These receptors bind to and are activated by several structurally dissimilar xenobiotics, fatty acids, and eicosanoids and are termed PPARs. Three related PPAR isotypes have been identified to date. They are termed PPARα, PPARβ/δ, and PPARγ[8,9]. Studies have documented the existence of human forms of PPARα (hPPARα)[10,11], PPARδ (hPPARδ )[9], and PPARγ (hPPARγ)[12]. The tissue distribution pattern of hPPARα mRNA is similar to that of the rat PPARα. In both species PPARα is highly expressed in brown adipose tissue, skeletal muscle, liver, heart, and kidney, but is expressed at low levels in the brain and lung[11,13]. The principal site of expression of PPARγ is the adipose tissue, but this receptor is also expressed, albeit at lower levels, in many other tissues and cell types such as the retina, some parts of the immune system, mammary and colonic epithelium[14]. PPARδ subtype is found in higher amounts than PPARα and PPARγ in almost all tissues examined, except the adipose tissue[15]. The chromosomal localization of the human and mouse PPAR genes has been defined[8]. The hPPARα gene was mapped on chromosome 22[8]. The hPPARγ gene is located on chromosome 3, and the hPPARδ has been assigned to chromosome 6[8]. In the mouse, PPARγ is located on chromosome 6, while PPARα and PPARδ are found on chromosomes 15 and 17, respectively[8].

Upon binding to their ligands, PPARs heterodimerize with retinoid X receptor prior to affecting gene transcription[16,17] (Fig. 1). There are three retinoid X receptor (RXR) genes coding for RXRα, RXRβ, and RXRγ [16,17]. PPARs appear to have preference for the RXRα subtype *in vivo*[16,17]. Consequently, RXRα-deficient mice do not respond to the gene-inductive effects of PPARα agonists[16,17].

### Relationship between PPARs and Cancer

A link between PPAR and cancer was first drawn after it became clear that agonists of PPARα caused a dramatic increase in the incidence of liver tumors in mice and rats[18,19,20]. The potential role of PPARγ in cancer formation in humans is controversial. A recent study implicated PPARγ in promotion and development of colon cancer[21]. Mice genetically predisposed to develop polyps in

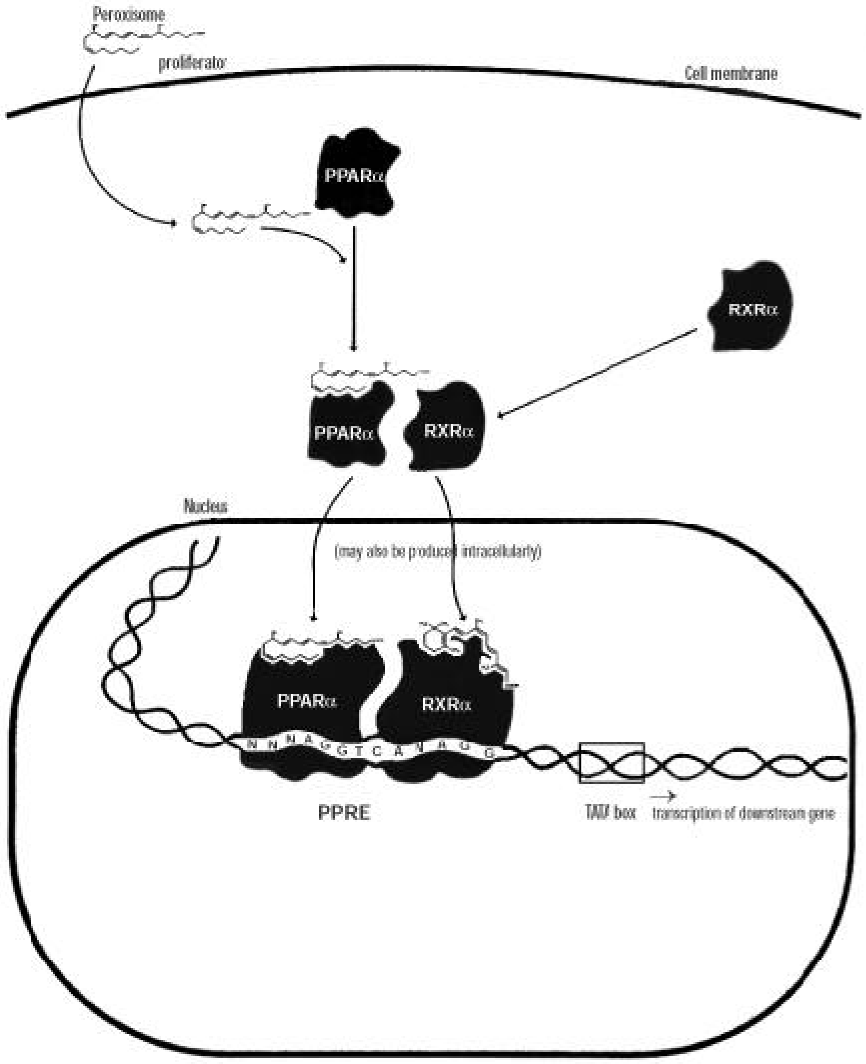

**FIGURE 1.** Schematic depiction of events involved in enhanced gene transcription by the heterodimers of PPAR and RXR. PPAR, peroxisome proliferator-activated receptor; RXR, retinoid X receptor.

the colon show an increased numbers of polyps when subjected to PPARγ agonists orally[21]. Conversely, it was demonstrated that human colon tumor cell lines both in culture and nude mice respond to PPARγ agonists with a reduced rate of growth and an increased degree of differentiation[22]. Consequently, further studies are needed to clarify the exact potential outcome from exposure to PPARγ agonists in humans. These agonists have been reported as useful for the differentiation in liposarcomas and breast tumors[23]. A recent report suggested that a reduction in the incidence of colorectal cancer in response to treatment with nonsteroidal anti-inflammatory agents may be due to the inhibition of PPARδ exerted by these drugs[24]. Confirmation of the involvement of PPARδ in colorectal cancer awaits more definitive studies using compounds specific to this PPAR subtype[9].

## AGING, PEROXISOMES, AND LIVER CANCER

Aged animals are more susceptible to the hepatocarcinogenic effect of peroxisome proliferators[25,26]. Long-term treatment with either of the peroxisome proliferating chemicals nafenopin or Wy-14,643 produced numerous hepatocellular adenomas and carcinomas in old male Wistar and Fischer 344 rats, while producing fewer changes in young animals[25,26].

## Effect of Aging on Peroxisomal Metabolism

Information on the relationship between aging and peroxisomal metabolism is scarce[20]. Studies point to a general decrease in peroxisomal function with aging[27]; however, several studies failed to document measurable differences in peroxisomal enzyme activities between young and old animals[25,28]. Apparent inconsistencies in the age-related effects on peroxisomal β-oxidation may have to do with species differences and the age of animals, as peroxisomal enzyme activities appear to change dramatically and abruptly at certain age points during development and aging[27].

The decline with aging in catalase activity in male Fischer 344 rats has been uniformly observed in various studies. In our laboratories, hepatic catalase activity declined by 40% between the ages of 4 and 100 weeks, with most of the decline (30%) observed between the ages of 50 and 100 weeks[29]. Similar findings were also observed by others[27] for both peroxisomal β-oxidation and catalase in both CSWV and OFi mice strains.

## Peroxisomal and Hepatocellular Proliferation in Aged Animals

It has been suggested that the carcinogenicity of PPARα agonists may be attributed to an increase in activities of peroxisomal oxidases[20]. Hydrogen peroxide, which is produced by these oxidases, may overwhelm cellular antioxidant defense mechanisms, react with cellular macromolecules and/or DNA, and potentially lead to cell damage. Alternatively, it has been suggested that of a greater significance to the hepatocarcinogenic effect produced by PPARα agonists is the increased cell replication produced by these chemicals[20]. Elevated cell replication is proposed to enhance the growth of hepatic lesions from spontaneously initiated cells into hepatic focal lesions; however, the relationship between hepatocarcinogenicity and either oxidative damage or cellular replication is not strong, as the correlation between these effects was found to be weak.

Aging also interferes with the ability of the liver to respond to peroxisome-proliferating chemicals[20,29]. While peroxisomal area relative to hepatocellular cytoplasmic area increased by about 7-fold in both 8- and 52-week-old rats, following 14 days of receiving daily doses of 200 mg/kg clofibrate in diet, it increased only by 4-fold in 117-week-old rats[29]. In the same rats, increases in hepatic peroxisomal β-oxidation showed a similar pattern, with increases approaching 15- and 11-fold in 8- and 52-week-old rats, but only 3-fold in the 117-week-old rats[20]. In our laboratory, perfluorooctanoic acid (PFOA) (Fig. 2) induced peroxisomal β-oxidation significantly in male Fischer 344 rats of varying age groups to different levels. After 48 h following 150 mg/kg PFOA, hepatic peroxisomal β-oxidation increased in 100-week-old rats to only about 60% of levels achieved in 10-week-old rats[28,29].

In contrast to the above findings with PFOA and in agreement with results reported in the literature[28], young and old male Fischer 344 rats given various concentrations of the peroxisome proliferator Wy-14,643 in the diet exhibited comparable hepatic peroxisomal β-oxidation activity. Similarly, a study in male Wistar rats showed that age did not seem to influence either basal or nafenopin-induced induction of peroxisomal β-oxidation[25]. In these long-term feeding studies[25,28], animals were given diets containing peroxisome proliferators for 22–59 weeks, rendering the age of young and old rats between 68 and 116 weeks at the conclusion of the experiment. Potentially, these are ages where responses to peroxisome proliferators are similar. In a more detailed study in our laboratories[20], striking results showing compound-specific effects were observed. While aging-related differences appeared to exist in the response of the liver to certain peroxisome proliferators, aging did not seem to influence the response to other peroxisome proliferators studied. The lack of uniformity in the response to various peroxisome proliferators

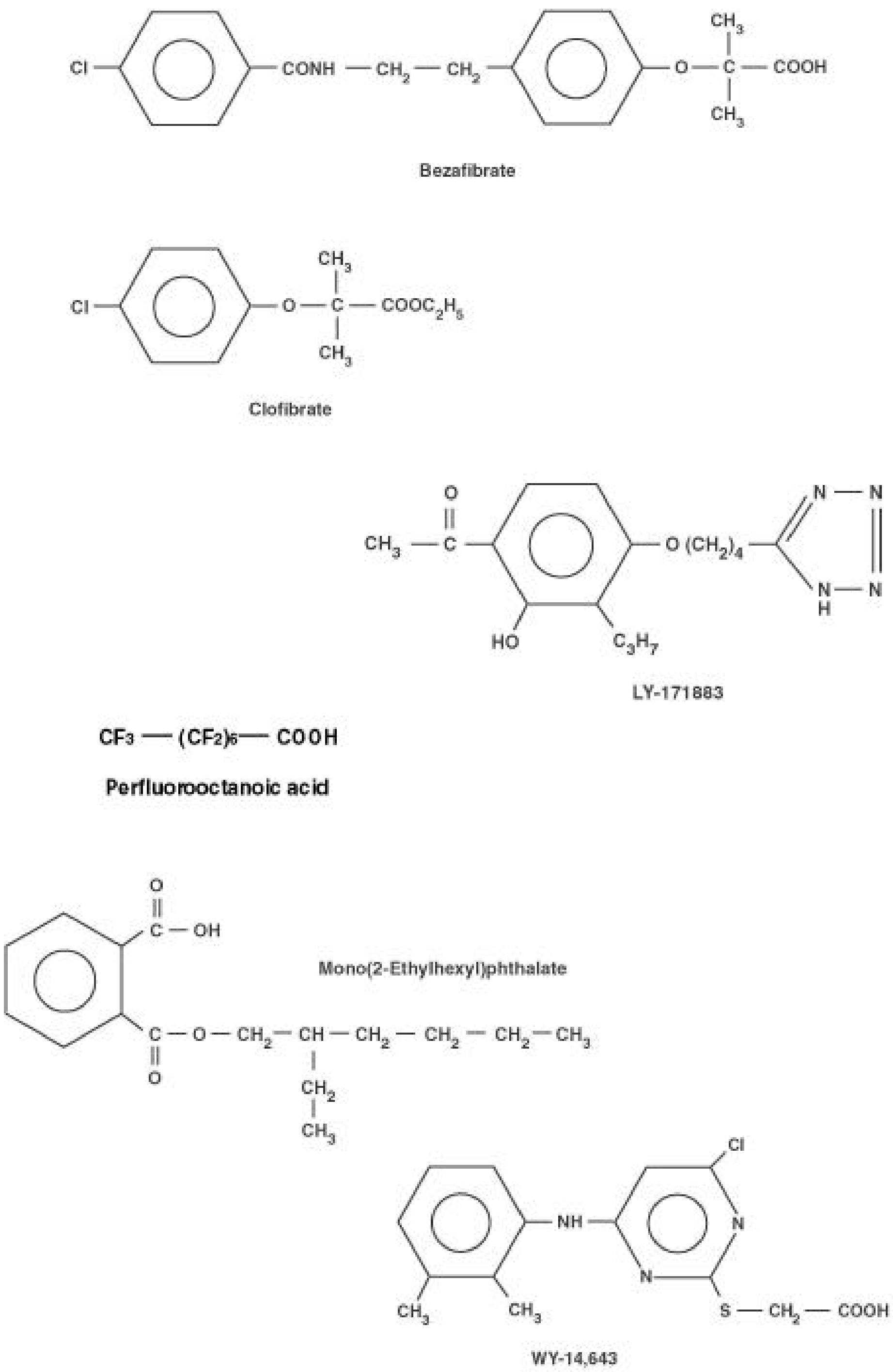

**FIGURE 2.** Chemical structures of representative PPAR agonists.

may be due to potential aging-related alterations in specific pathways involved in the metabolism of specific proliferators. In other words, peroxisomal signal transduction pathways are most likely altered in old animals, as evidenced by the significant differences observed between young and old animals in response to the nonmetabolizable PFOA.

Cyclin (PCNA) is a highly conserved 36-kD acidic nuclear protein which is directly involved in DNA synthesis as a cofactor for DNA polymerase δ, as well as in unscheduled DNA synthesis[30].

The cyclin gene is transcribed efficiently in both quiescent and proliferating cells, but its mRNA accumulates only in proliferating cells[31]. The absence of stable cyclin mRNA in quiescent cells is associated with the presence of intron 4 in the gene; removal of this intron leads to high levels of accumulation of cyclin mRNA[31]. Accumulation of cyclin mRNA and the synthesis of high levels of the protein product are stimulated by growth factors[31].

Quantification of cyclin mRNA in livers of various animal groups revealed no age-dependent differences in the constitutive levels of this marker of cell proliferation (Fig. 3). Furthermore enhanced hepatic expression of this mRNA by the known peroxisome proliferator Wy-14,643 was comparable in all animal age groups studied (Fig. 3). This finding casts doubt on the importance of hepatocellular proliferation as a pivotal factor in the enhanced susceptibility of the aged liver to the carcinogenic effect of PPAR agonists.

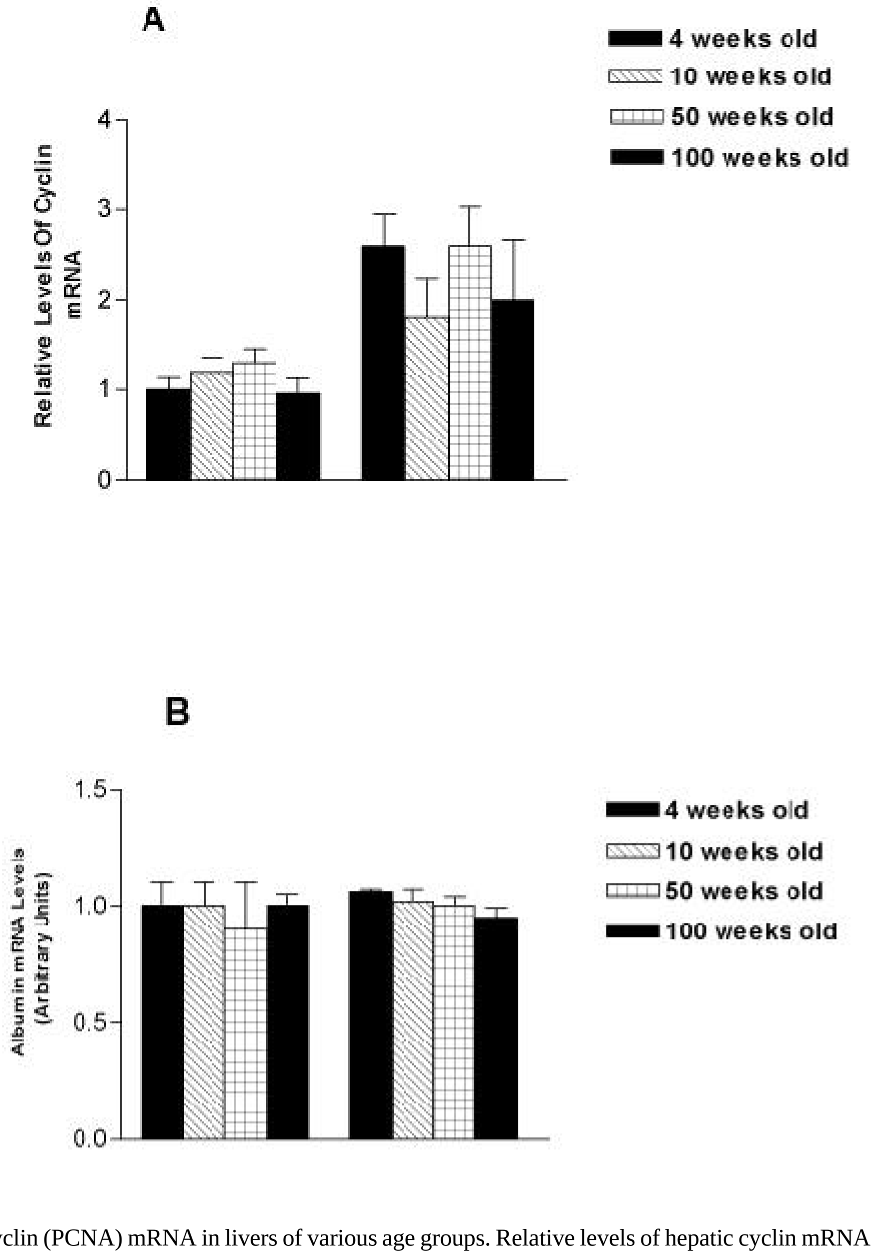

**FIGURE 3.** Cyclin (PCNA) mRNA in livers of various age groups. Relative levels of hepatic cyclin mRNA in control (left bar group) and Wy-14,643-treated rats (right bar group) normalized against albumin mRNA were determined by RT-PCR. Data are means ±SEM of 4-6 animals per group.

## Aging, Apoptosis, and Response to PPAR Agonists

The enhanced hepatocarcinogenic susceptibility of old animals to PPAR agonists did not appear to result from enhanced hepatic oxidative stress or uncontrolled hepatocellular proliferation. Consequently, our attention was drawn to investigate other factors that may play a crucial role in rendering the aged liver more susceptible to the carcinogenic effect of these chemicals.

Studies have shown that peroxisome proliferators suppress apoptosis in the liver in a process mediated by PPARα[32,33]. Apoptosis appears to be a safeguard to prevent cells with damaged DNA from progressing to a tumor[34]. In a primary culture of rat hepatocytes and in a FaO rat hepatoma cell line, apoptosis was induced by TGFB1, an effect that was significantly reduced by the coadministration of the peroxisome proliferator nafenopin[35]. Furthermore, nafenopin suppressed hepatic apoptosis *in vivo*, and its withdrawal resulted in a 100- to 200-fold elevation in apoptosis[35]. Hepatocytes generated during nafenopin-induced hyperplasia were not the same ones that underwent apoptosis upon nafenopin withdrawal[35]. Hepatocytes resistant to apoptotic death may represent preferential targets for promotion to tumors by peroxisome proliferators, and hence suppression of apoptosis may play a role in the hepatocarcinogenicity of this class of nongenotoxic compounds.

Because hepatocytes resistant to apoptotic death may proceed to become malignant, we hypothesized that the aging liver may be more sensitive to the antiapoptotic effect of peroxisome proliferators. This allows aged hepatocytes with DNA damage to progress to a tumor[32]. We consequently quantified the effect of the prototype peroxisome proliferator Wy-14,643 on the mRNA levels of various elements of the apoptotic machinery, spanning death receptors to upstream and downstream caspases as well as two antagonistic members of the Bcl-2 family.

### *Bcl-2 Family of Proteins*

Members of the Bcl-2 protein family are key regulators of apoptosis, with various members of this family exhibiting pro- or antiapoptotic activity[36]. Relative abundance of opposing members of this family of proteins may act as a rheostat for the suicide program[36]. In our laboratories the PPAR agonist Wy-14,643 decreased the antiapoptotic Bcl-$x_L$ mRNA in all age groups except in 10-week-old rats where levels were slightly increased (Fig. 4). Conversely, Bax, a proapoptotic member of the Bcl-2 family, plummeted significantly in all groups, except in 10-week-old rats where it was remarkably elevated in response to Wy-14,643 (Fig. 4). These data suggest that while peroxisome proliferators are known to inhibit apoptosis, these chemicals may indeed enhance apoptosis only in young mature animals. Indeed, a closer look at the literature revealed a recent report showing a doubling of liver apoptotic cell number in young adult mice treated with Wy-14,643[37]. This observation is in contrast to the prevailing dogma, and likely that was the reason this finding was not elaborated on in that report[37].

### *Caspase Family of Proteases*

Caspases, a family of proteases, constitute a central component of the cellular apoptotic machinery[38,39]. These enzymes participate in a cascade that is triggered in response to proapoptotic signals, leading to cell death[38,39]. Deficiency in Caspase-1 renders cells resistant to the apoptotic effect of Fas/APO-1[39]. Interestingly, Wy-14,643 remarkably diminished the mRNA levels of both Caspase-1 and Fas in all age groups, except 10-week-old rats (Fig. 4). Only in this age group, WY-14,643 increased Fas mRNA by 50% and did not alter that of Caspase-1 (Fig. 4). Again, this effect is in agreement with our hypothesis stipulating that peroxisome proliferators may induce an apoptotic response in young adult animals, while diminishing this response in both juvenile and senescent animals.

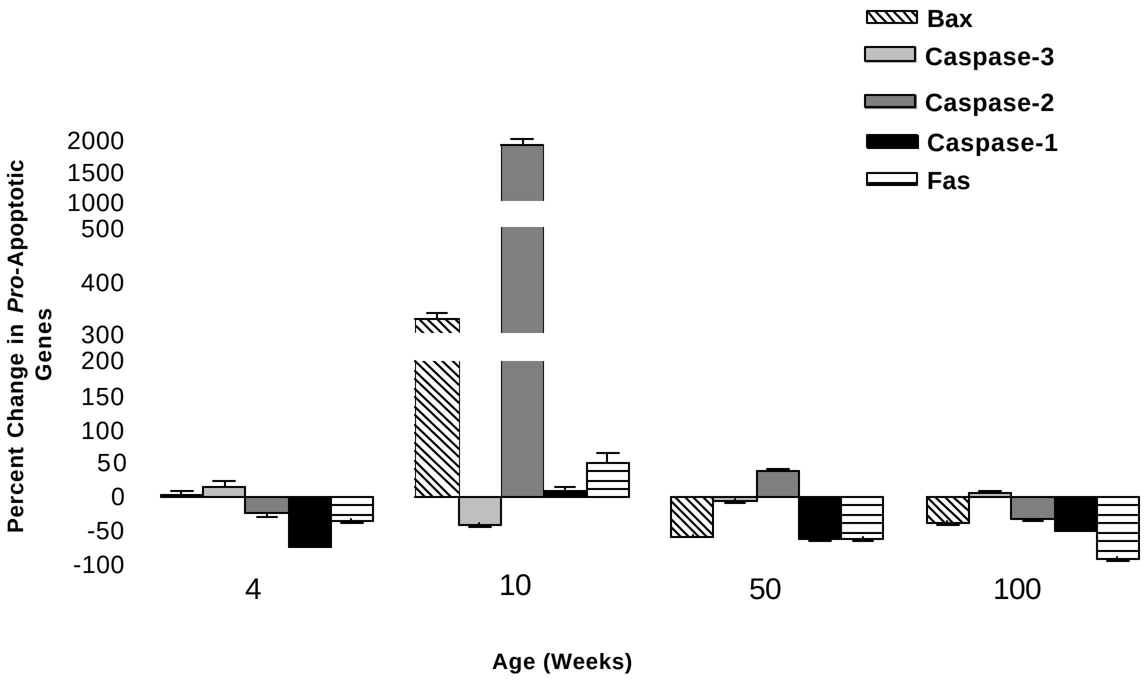

**FIGURE 4.** Effect of age on elements of the hepatic proapoptotic machinery. mRNA of various proapoptotic factors were quantified by ribonuclease protection assay and normalized against L 32, and percent change from respective untreated values were calculated. Data are means ± SEM of three animals per group.

Our experiments further yielded a remarkable observation on age-related modulation of peroxisome proliferator-induced effects on the expression of hepatic Caspase-2 mRNA. Levels of Caspase-2 mRNA plummeted in livers of juvenile (4-week-old) and old (100-week-old) rats, but increased by an astounding 19-fold in the livers of 10-week-old rats (Fig. 4). There was a modest increase of 36% in the livers of 50-week-old rats (Fig. 4**)**. This finding heightens the paradoxical effect of age on the sensitivity to proapoptotic signals. This is an important finding in light of the fact that overexpression of Caspase-2 has been reported to induce apoptosis in a variety of cell lines and upregulation of Caspase-2 mRNA was observed under conditions which stimulated apoptosis[38,39].

The impact of age on Wy-14,643-induced effects on the downstream effector Caspase-3 was intriguing. The mRNA of this enzyme was reduced by 40% only in the livers of the apoptotic-prone 10-week-old rat group, in the absence of significant effects in all other animal groups (Fig. 4). Activation of upstream caspases such as Caspase-2 observed in these livers (Fig. 4) results in the activation of Caspase-3[38,39]; therefore, a decrease in Caspase-3 mRNA in the apoptotic-prone livers (Fig. 4) may reflect an over-utilization of this caspase. In other words, the robust increase in the mRNA of Caspase-2 in apoptotic-prone livers (Fig. 4) may be contributing to the inability of these cells to maintain high levels of Caspase-3 mRNA levels in the face of continued consumption of the enzyme through its activation by proteolysis[38,39].

## CONCLUSIONS

The enhanced susceptibility of old animals to the hepatocarcinogenic effect of PPAR activators, many of which exist in the environment as pollutants, does not appear to result from increased hepatic oxidative stress or uncontrolled hepatocellular proliferation in senescent animals. Novel findings from our laboratories show that the aged liver is exceedingly sensitive to the antiapoptotic effect of PPAR agonists (preliminary, unpublished data). Furthermore, the PPAR agonist Wy-14,643 diminished elements of the proapoptotic machinery (e.g., Bax, caspases, or fas) significantly in the aged liver, while increasing crucial elements of this machinery in younger animals. Our data suggest that PPAR agonists suppress the ability of the aged liver to purge itself of already transformed cells via apoptosis, allowing these cells to progress to malignancy. The proapoptotic effect of these chemicals in livers of young adult rats may contribute to the diminished susceptibility of these animals to the hepatocarcinogenic effect of peroxisome proliferators.

## ACKNOWLEDGMENTS

Studies in our laboratories are supported by NIH AG 18479.
